# Renal Tubular Cells from Hibernating Squirrels are Protected against Cisplatin Induced Apoptosis

**DOI:** 10.1155/2020/6313749

**Published:** 2020-08-04

**Authors:** Swati Jain, Alkesh Jani

**Affiliations:** ^1^University of Colorado, Denver, CO, USA; ^2^Denver Veterans Affairs Medical Center, Denver, CO, USA

## Abstract

Hibernating 13-lined ground squirrels are characterized by tolerance of severe hypothermia and hypoperfusion during torpor, followed by periodic warm reperfusion during IBA, conditions which are lethal to nonhibernating mammals. The aim of the present study was to determine whether protection from apoptosis was specific to torpor arousal cycles during hibernation or will also apply to cisplatin treatment on squirrel renal tubular cells (RTECs) that were procured during hibernation. Squirrel and mouse RTECs were treated with cisplatin, a potent inducer of RTEC apoptosis. Squirrel RTECs subjected to cisplatin had significantly less apoptosis, no cleaved caspase-3, and increased XIAP, pAkt, and pBAD versus mouse RTECs. To determine whether XIAP and Akt1 are necessary for RTEC protection against cisplatin induced apoptotic cell death, gene expression of Akt1 or XIAP was silenced in squirrel RTECs. Squirrel RTECs deficient in Akt1 and XIAP had increased apoptosis and cleaved caspase-3 when treated with cisplatin. Our results thus demonstrates that 13-lined ground squirrel RTECs possess intrinsic intracellular mechanisms by which they protect themselves from apoptotic cell death. Cisplatin induced acute kidney injury (AKI) may be avoided in cancer patients by employing mechanisms used by squirrel RTECs to protect against cisplatin induced tubular cell apoptosis.

## 1. Background

**Figure 1:**
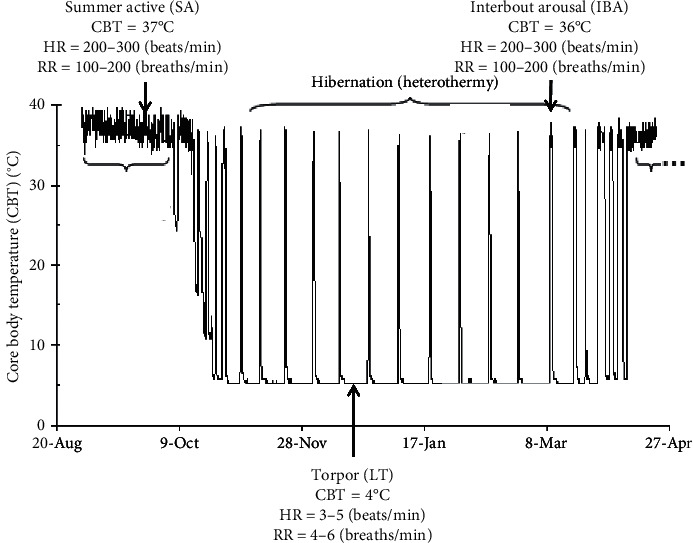
Representative core body temperature (CBT), heart rate, and respiratory rate during summer (SA), torpor (LT), and interbout arousal (IBA). CBT nadirs at ∼4°C for several days during torpor, at which time heart and respiratory rates decline from a summer-time level of 200–300 to 3–5 beats/min and respiratory rate from 100–200 to 4–6 breaths/min. During periodic IBA, CBT returns to ∼37°C for ∼12 hours, and heart rate and respiratory rate return to normal. Thereafter, the CBT falls again as the hibernator re-enters torpor. The cycle of torpor and IBA is repeated several times during one winter season suggesting hibernating kidneys use natural mechanism to avoid damage by cold ischemia followed by warm reperfusion.

The 13-lined ground squirrel (*Ictidomys tridecemlineatus*) is a mammalian hibernator that undergoes extreme reductions in core body temperature (CBT) from 38°C to ∼4°C for up to *18 days* during a period named torpor [1]. During torpor, the ground squirrel heart rate is reduced from a summer-time level of 300 to ∼3 beats/min (∼1% of normal) [2] and the respiratory rate is decreased from 200 to ∼4 breaths/min (∼2% of normal) [3], conditions that are lethal to nonhibernators [4]. Torpor is periodically interrupted by rewarming to 38°C and warm reperfusion of organs for ∼24 hours during interbout arousal (IBA) [1]. The functional reason for arousal is not known [1, 5]. Thereafter, the hibernator re-enters torpor and cycles through torpor-arousal ∼20 times in a winter season [1] (Figure 1).

Hibernators are thus characterized by tolerance of severe hypothermia followed by warm reperfusion. These 13-lined ground squirrels are of great interest in understanding their remarkable tolerance to extreme condition compared to nonhibernators. To overcome the limited supply of these squirrels per season, exclusive and challenging housing condition, and the lack of genetic manipulated squirrels, we have isolated and immortalized the renal tubular epithelial cells (RTECs) from hibernating ground squirrel through the expression of telomerase reverse transcriptase (TERT) protein as previously described [6].

We have previously shown that, remarkably, kidneys of hibernating 13-lined ground squirrels during IBA have completely normal renal function and no renal tubular cell (RTEC) apoptosis despite warm reperfusion after prolonged CI of several days duration, far in excess of that tolerated by human donor kidneys. Also, ground squirrel RTECs are protected against apoptosis subjected to prolonged in vitro cold storage and rewarming [6, 7]. In this study, we sought to determine whether protection from apoptosis was specific to torpor arousal or was a nonspecific response that would occur in response to any apoptotic stimuli. In the current study, we procured cortical renal tubular cells (RTECs) from hibernating 13-lined ground squirrel kidneys and subjected them to treatment with the chemotherapeutic agent cisplatin, a potent inducer of renal tubular cell death [8]. We hypothesized that the ground squirrel RTECS demonstrated intrinsic protective mechanisms that would protect them from apoptosis during treatment with cisplatin in a setting distinct from torpor arousal.

## 2. Methods

### 2.1. Reagents and Antibodies

Rabbit polyclonal antibodies against cleaved caspase-3, XIAP, pAkt (ser473), pBAD (ser136), Akt1, and actin were purchased from Cell Signaling Technology (Boston, MA, USA). DeadEnd™ Colorimetric TUNEL assay kit was purchased from Promega (Madison, WI, USA). XIAP siRNA (Catalog ID: 4390824) and siRNA negative control (Catalog ID: 4390843) were purchased from Life Technologies (Grand Island, NY, USA). Akt1 shRNA lentiviral particles (Catalog ID: 29195 v), polybrene, and puromycin dihydrochloride were purchased from Santa Cruz Biotechnologies (Dallas, TX, USA).

### 2.2. Cell Culture

Renal tubular cells of hibernating ground squirrel and mouse renal tubular cells (used as a positive control from a non-hibernating species) and were cultured in Dulbecco's modified Eagles medium (DMEM)/F-12 50/50 medium and RPMI 1640 medium respectively, supplemented with 10% fetal bovine serum (FBS), 100 units/ml penicillin, and 100 *µ*g/ml streptomycin at 37°C in a humidified atmosphere with 5% CO_2_ and fed with fresh medium at intervals of 48 h.

### 2.3. Cisplatin Treatment of Cells

Experiments were performed with cells grown to 70–80% confluence. For cisplatin treatment, mouse and squirrel RTECs were incubated with vehicle (DMSO) or 10 *µ*M and 50 *µ*M of cisplatin for 24 h.

### 2.4. Cell Lysis and Western Blot Analysis

Cells were lysed in RIPA buffer for 10 minutes on ice and then subjected to sonication for 10 s. The lysate was centrifuged at 14,000 g for 10 min at 4°C. The supernatant was collected and protein concentration was determined by Bradford assay. Equal amounts of protein were separated on sodium dodecyl sulfonate- (SDS-) PAGE, transferred onto a nitrocellulose membrane, blocked, and incubated with primary antibodies overnight at 4°C. After washing, the membrane was incubated with a horseradish peroxidase-labeled secondary antibody. ECL was used as a method of detection. Each membrane was stripped and reprobed with anti *β*-actin antibody, to verify equal protein loading. Blots for every protein were performed in 3 different sets of experiments to validate the results.

### 2.5. Transfection of XIAP Small Interfering RNA (siRNA)

XIAP siRNA was used to reduce the expression of XIAP in squirrel RTECs. Briefly, squirrel RTECs were plated in 60 mm dishes in growth medium without antibiotics such that they were 80–85% confluent at the time of transfection. Cells were transfected for 24 hours with XIAP 100 pmol siRNA or control nonspecific siRNA with the transfection reagent lipofectamine2000™. Cells were further treated with cisplatin and after exposing to apoptotic stimuli cells were harvested and analyzed for the expression of proteins.

### 2.6. Transfection of Akt1 Short Hairpin RNA (shRNA)

Akt1 shRNA was used to make a stable colony of squirrel RTECs in which gene expression of Akt1 was reduced. Briefly, squirrel RTECs were plated in 60 mm dishes in growth medium without antibiotics to achieve 50% confluence at the time of transfection. Polybrene at a final concentration of 5 *µ*g/ml was added before adding lentiviral particles. Cells were then infected by adding Akt1 shRNA lentiviral particles and incubated overnight. On the following day, the cells were split in a 1 : 3 ratio and further incubated for 48 hours in complete growth medium. To select stable clones expressing Akt1 shRNA, puromycin dihydrochloride selection was used. Complete medium with puromycin was replaced every 3–4 days until resistant colonies were identified. Several colonies were picked and expanded and assayed for stable shRNA expression. Colonies with 60–70% knockdown expression of Akt1 were picked for further study. Cells derived from a colony with a stable knockdown of Akt1 (60–70%) colony were further treated with cisplatin and after exposing to apoptotic stimuli cells were harvested and analyzed for the expression of proteins.

### 2.7. TUNEL Assay

Cells were grown in chamber slides to perform a TUNEL assay. Apoptosis was assessed both by TUNEL assay and morphologically for specific detection and quantitation of apoptotic cells. TUNEL assay was performed according to manufacturer's directions and apoptotic RTECs characterized by round, shrunken, and pyknotic dark brown nuclei. Apoptotic nuclei were counted in five power fields in a blinded fashion using a light microscope at 40X.

### 2.8. Statistical Analyses

All data and results presented were confirmed in at least three independent experiments. All values are expressed as mean ± SEM. For multiple comparisons, data were analyzed by ANOVA using Tukey's Multiple Comparison Test. A *P* value of <0.05 was considered significant.

## 3. Results

### 3.1. Ground Squirrel RTECs Subjected to Cisplatin Have Decreased Active Caspase-3 Protein Expression and Are Protected from Apoptosis

**Figure 2:**
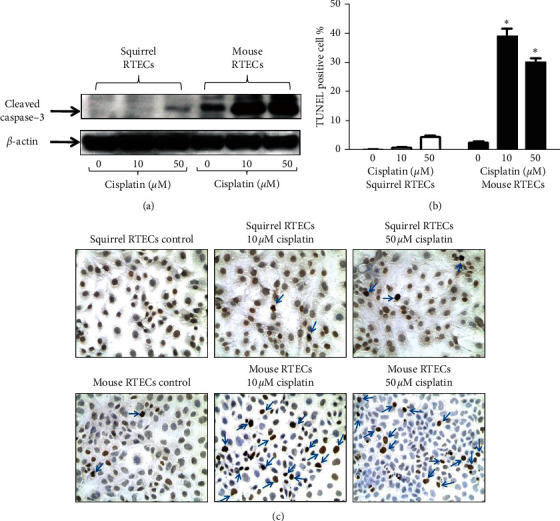
(a) Representative blot shows cleaved caspase-3 protein expression is increased in mouse RTECs treated with 10 *µ*M and 50 *µ*M cisplatin compared to control mouse RTECs, control squirrel RTECs, and squirrel RTECs treated with 10 *µ*M and 50 *µ*M cisplatin. (b) TUNEL + apoptotic cells are significantly increased in mouse RTECs treated with 10 *µ*M and 50 *µ*M cisplatin versus control mouse RTECs, control squirrel RTECs, and cisplatin-treated squirrel RTECs (^*∗*^*p* < 0.0001 versus mouse and squirrel RTECs treated with 0 *µ*M cisplatin (controls) and squirrel RTECs treated with 10 *µ*M and 50 *µ*M cisplatin, *n* = 3). (c) Representative pictures of TUNEL staining in squirrel and mouse RTECs treated with cisplatin. *β*-actin is used as a protein loading control. Vehicle treated cells are referred to as controls. A densitometry and statistical analysis of immunoblot is given in Supplementary Figure S1.

Ground squirrel RTECs and mouse RTECs were subjected cisplatin treatment in increasing doses of 10 *µ*M and 50 *µ*M. Ground squirrel and mouse RTECs treated with vehicle were used as controls. There was no significant increase in expression of cleaved caspase-3 in ground squirrel RTECs subjected to increasing doses of cisplatin versus control ground squirrel RTECs. In contrast, cleaved caspase-3 protein expression was significantly increased in mouse RTECs treated with cisplatin versus mouse RTECs treated with vehicle (control) and squirrel RTECs treated with vehicle (control) or with cisplatin (Figure 2(a) and Supplementary data, Figure S1). Morphological examination of squirrel RTECs was combined with TUNEL staining for assessment of apoptosis. There was no significant difference in percent apoptosis in squirrel RTECs treated with cisplatin compared to controls. In contrast, the number of apoptotic cells were significantly increased in mouse RTECs treated with cisplatin versus mouse RTECs treated with vehicle (control) and squirrel RTECs treated with vehicle (control) or with cisplatin (Figures 2(b) and 2(c)).

### 3.2. Increased Expression of Antiapoptotic Proteins Is Associated with Protection of Ground Squirrel RTECs during Cisplatin Induced Apoptosis

**Figure 3:**
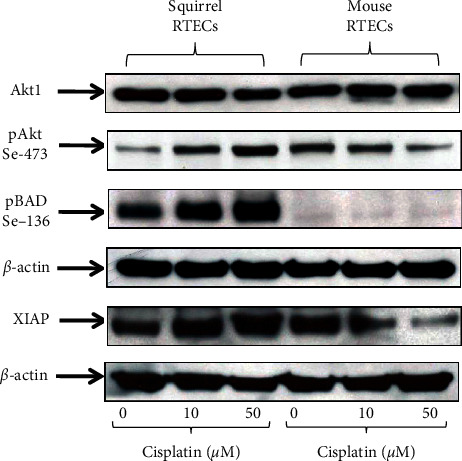
Representative blots show increased expression of pAkt (ser473), pBAD (ser136), and XIAP protein in squirrel RTECs on treatment with 10 *µ*M and 50 *µ*M cisplatin versus control squirrel RTECs. In contrast, mouse RTECs have reduced expression of pAkt (ser473), pBAD (ser136), and XIAP with cisplatin treatment compared to control mouse RTECs. *β*-actin is used as a protein loading control. Vehicle treated cells are referred to as controls. Densitometry and statistical analyses of immunoblots are given in Supplementary Figure S2.

To determine whether the protection of ground squirrel RTECs from caspase-3 activation and apoptotic cell death during cisplatin treatment was associated with an increase in antiapoptotic proteins, we examined the expression of XIAP, pAkt (Ser473), and pBAD (Ser136) in RTECS treated with cisplatin. XIAP, pAkt (ser473), and pBAD (ser136) protein expression was significantly increased in squirrel RTECs treated with cisplatin versus squirrel RTECs treated with vehicle and decreased in mouse RTECs-treated cisplatin versus mouse RTECs treated with vehicle (Figure 3 and Supplementary data, Figure S2).

### 3.3. Akt1 Deficient Ground Squirrel RTECs Undergo Apoptosis when Subjected to Cisplatin Treatment Compared to Untreated Wild Type Ground Squirrel RTECs

**Figure 4:**
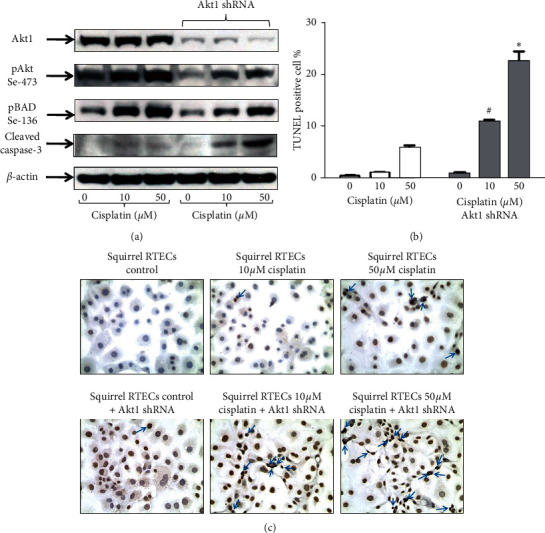
(a) Representative blots show Akt1 shRNA-treated squirrel RTECs have significantly reduced expression of Akt1. Akt1 deficient squirrel RTECs treated with cisplatin have decreased protein expression of pAkt (ser473), pBAD (ser136), and increased expression of cleaved caspase-3 versus controls and wild type squirrel RTECs treated with cisplatin. (b) TUNEL + apoptotic cells are significantly increased in Akt1 deficient squirrel RTECs treated with 10 *µ*M and 50 *µ*M cisplatin versus wild type squirrel RTECs, untreated Akt1 deficient squirrel RTECs, and wild type squirrel RTECs treated with cisplatin. (^#^*p* < 0.001 and ^*∗*^*p* < 0.0001 versus wild type and Akt1 deficient squirrel RTECs treated with 0 *µ*M cisplatin (controls) and wild type squirrel RTECs treated with 10 *µ*M and 50 *µ*M cisplatin, *n* = 3). (c) Representative pictures of TUNEL staining in wild type and Akt1 negative squirrel RTECs treated with cisplatin. *β*-actin is used as a protein loading control. Vehicle treated cells are referred to as controls. Densitometry and statistical analyses of immunoblots are given in Supplementary Figure S3.

To determine whether pAkt (Ser473) and pBAD (Ser136) were required in protecting squirrel RTECs from cisplatin induced apoptosis, we reduced Akt1 gene expression in ground squirrel RTECs by 60–70% using Akt1 shRNA (Figure 4(a) and Supplementary data, Figure S3(i)). Akt1 deficient ground squirrel RTECs treated with cisplatin had (a) significantly decreased pAkt (Ser473) expression (Figure 4(a) and Supplementary data, Figure S3(ii)), (b) significantly reduced pBAD (Ser136) expression (Figure 4(a) and Supplementary data, Figure S3(iii)), (c) significantly increased cleaved caspase-3 protein expression (Figure 4(a) and Supplementary data, Figure S3(iv)), and (d) significantly increased apoptosis versus untreated wild type ground squirrel RTECs (*p* < 0.001) (Figures 4(b) and 4(c)).

### 3.4. XIAP Deficient Ground Squirrel RTECs Undergo Apoptosis when Subjected to Cisplatin Treatment Compared to Untreated Wild Type Ground Squirrel RTECs

**Figure 5:**
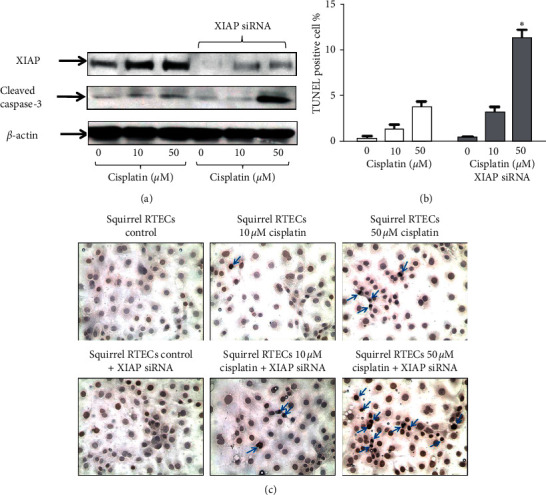
(a) Representative blots show reduced expression of XIAP in squirrel RTECs treated with XIAP siRNA. Cisplatin and XIAP siRNA-treated squirrel RTECs have decreased protein expression of XIAP and increased expression of cleaved caspase-3 compared to controls and wild type cisplatin-treated squirrel RTECs. (b) TUNEL + apoptotic cells are increased significantly in 50 *µ*M cisplatin-treated XIAP deficient squirrel RTECs versus wild type and XIAP deficient controls and wild type cisplatin-treated squirrel RTECs (^*∗*^*p* < 0.0001versus wild type and XIAP deficient squirrel RTECs treated with 0 *µ*M cisplatin (controls), wild type squirrel RTECs treated with 10 *µ*M and 50 *µ*M cisplatin, and XIAP deficient squirrel RTECs treated with 10 *µ*M cisplatin, *n* = 3). (c) Representative pictures of TUNEL staining in wild type and XIAP negative squirrel RTECs treated with cisplatin. *β*-actin is used as a protein loading control. Vehicle treated cells are referred to as controls. Densitometry and statistical analyses of immunoblots are given in Supplementary Figure S4.

To determine the role of XIAP in protecting ground squirrel RTECS from apoptosis during treatment with cisplatin, we knocked down XIAP gene expression using siRNA. XIAP deficient ground squirrel RTECs treated with cisplatin had (a) significantly decreased XIAP expression (Figure 5(a) and Supplementary data, Figure S4(i)), (b) significantly increased cleaved caspase-3 protein expression (Figure 5(a) and Supplementary data, Figure S4(ii)), and (c) significantly (*p* < 0.001) increased apoptotic cells versus untreated wild type ground squirrel RTECs (Figures 5(a) and 5(b)).

## 4. Discussion

In this study, we sought to discover whether 13-lined ground squirrel RTECs are protected from apoptotic cell death during treatment with cisplatin, a potent inducer of apoptosis [8]. Studies of the protective mechanisms employed by ground squirrels may suggest novel clinical approaches for the prevention and treatment of apoptosis. We chose to use cisplatin in this study for two reasons: (1) cisplatin is a known potent inducer of renal tubular cell apoptosis; (2) cisplatin is a known cause of acute kidney injury and demonstration of protection of RTEC apoptosis from cisplatin might lead to novel therapies for cisplatin induced AKI [8, 9].

We focused on upstream mediators of capase-3 activation, namely, X-linked inhibitor of apoptosis protein (XIAP) and Phospho Akt (ser473). XIAP is a naturally occurring inhibitor of caspase-3 [10]. XIAP belongs to the Inhibitor of apoptosis protein (IAP) family, whose members bind and inhibit caspase-3, caspase-7, and/or caspase-9, but not caspase-8 [11]. IAP family members are classified according to the presence of baculovirus IAP repeat (BIR) domains [12]. Class 1 IAPs, including XIAP, contain a RING finger domain and homologous BIR domains [12]. The BIR 2 domain of XIAP inhibits effector caspase-3 and caspase-7, while the BIR 3 domain of XIAP inhibits the initiator caspase-9 [13]. Loss of XIAP is implicated in cisplatin induced RTEC apoptosis [14].

Akt has been shown to prevent apoptotic death of a variety of cell types induced by a number of stimuli, including DNA damage, withdrawal of growth factors, and loss of cell adhesion [15]. pAkt (ser473) exerts its antiapoptotic function in part via phosphorylating BAD at serine 136 to its inactive form [15]. PhosphoBAD (ser136) is an important negative regulator of the antiapoptotic protein Bcl-xL [16, 17], which stabilizes the mitochondrial membrane and prevents the release of the proapoptotic cytochrome-C [18].

Squirrel cortical tubular cells had significantly reduced tubular cell apoptosis following treatment with cisplatin, which was associated with significantly decreased caspase-3 protein expression and increased protein expression of XIAP, pAkt (ser473), and pBAD (ser136), versus mouse RTECs controls. To demonstrate that XIAP and phosphorylated Akt1 were required for protection from apoptosis, we used a gene silencing approach against XIAP and Akt. Squirrel cortical cells treated with siRNA against XIAP had increased protein expression of cleaved caspase-3 and a significant increase in TUNEL + apoptotic cells versus untreated squirrel cortical cells following treatment with cisplatin. Similarly, treatment of squirrel cortical cells with shRNA against Akt1 resulted in ∼60–70% decrease in Akt1 protein expression, significantly less pAkt (ser473) pBAD (ser136), and significantly more cleaved caspase-3 and TUNEL + apoptotic cells following treatment with cisplatin. Thus, squirrel cortical cells employ the antiapoptotic proteins XIAP and phosphorylated Akt to prevent apoptosis induced by cisplatin.

Our findings suggest that 13-lined ground squirrel RTECs possess intrinsic intracellular mechanisms by which they protect themselves from apoptotic cell death. Survival during torpor arousal therefore may entail more complex mechanisms that simply match physiological supply and demand [6]. Our findings also suggest that protection from tubular cell apoptosis during treatment with cisplatin for cancer may be achieved by upregulation of XIAP and Akt. Cisplatin is a significant cause of acute kidney injury [9] in patients receiving the drug as part of a chemotherapy regimen. Employing similar protective mechanisms used by squirrel RTECs to prevent cisplatin induced tubular cell apoptosis may offer a novel therapeutic approach to preventing acute kidney injury (AKI) in patients who require cisplatin to treat a malignancy. A limitation to this approach, however, is that upregulation of XIAP is associated with cisplatin resistance in head and neck cancer [19] and increased Akt expression and activity are associated with acquired cisplatin resistance in lung cancer cells [20]. Therefore, renal protection during cisplatin therapy would require targeted upregulation of XIAP and pAkt in renal tubular cells and not in nonkidney cancer cells for before such an approach could be applied in a clinical setting. There are currently efforts underway to create targeted, organ-specific delivery of therapeutic drugs such as affinity-based physical targeting that employs peptides or antibodies as an “address tag” [21] or hydrogels, which can be injected under the kidney capsule and deliver therapeutic drugs [22]. Further studies are necessary to determine whether organ-specific targeted therapy can provide local kidney protection during systemic cisplatin chemotherapy.

In conclusion, our findings suggest that squirrel RTECs are protected from cisplatin induced apoptosis in association with upregulation of XIAP, pAkt (ser473), and pBAD (ser136). By studying tolerance of squirrel RTECs against cisplatin treatment, we have identified therapeutic targets that may lead to protection against cisplatin induced AKI.

## Data Availability

The data used to support the findings of this study are available from the corresponding author upon request.

## Abbreviations

RTECs: Renal tubular cells
AKI: Acute kidney injury
IBA: Interbout arousal.

## Supplementary Materials

Supplementary Materials Supplementary Figure S1: cleaved caspase-3 protein expression is increased in mouse RTECs treated with 10 *µ*M and 50 *µ*M cisplatin compared to control mouse RTECs, control squirrel RTECs, and squirrel RTECs treated with 10 *µ*M and 50 *µ*M cisplatin (^*∗*^*p* < 0.0001 versus mouse and squirrel RTECs treated with 0 *µ*M cisplatin (controls), and squirrel RTECs treated with 10 *µ*M and 50 *µ*M cisplatin, *n* = 3). Supplementary Figure S2: pAkt (ser473), pBAD (ser136), and XIAP protein expression are increased in squirrel RTECs on treatment with 10 *µ*M and 50 *µ*M cisplatin versus control squirrel RTECs. In contrast, mouse RTECs have reduced expression of pAkt (ser473), pBAD (ser136), and XIAP with cisplatin treatment compared to control mouse RTECs. (i) Akt1 (^*∗*^*p* < 0.01versus squirrel RTECs treated with 50 *µ*M cisplatin, *n* = 3). (ii) pAkt (ser473) (^*∗*^*p* < 0.001versus mouse and squirrel RTECs treated with 0 *µ*M cisplatin (controls), squirrel RTECs treated with 10 *µ*M cisplatin and mouse RTECs treated with 10 *µ*M and 50 *µ*M cisplatin; ^#^*p* < 0.0001 versus squirrel RTECs treated with 0 *µ*M cisplatin (control) and mouse RTECs treated with 50 *µ*M cisplatin, *n* = 3). (iii) pBAD (ser136) (^*∗*^*p* < 0.0001versus mouse and squirrel RTECs treated with 0 *µ*M cisplatin (controls), squirrel RTECs treated with 10 *µ*M cisplatin, and mouse RTECs treated with 10 *µ*M and 50 *µ*M cisplatin; ^#^*p* < 0.0001 versus mouse and squirrel RTECs treated with 0 *µ*M cisplatin (controls), and mouse RTECs treated with 10 *µ*M and 50 *µ*M cisplatin; ^$^*p* < 0.0001 versus mouse RTECs treated with 0 *µ*M cisplatin (control), and mouse RTECs treated with 10 *µ*M and 50 *µ*M cisplatin, *n* = 3). (iv) XIAP (^*∗*^*p* < 0.0001versus mouse and squirrel RTECs treated with 0 *µ*M cisplatin (controls), squirrel, and mouse RTECs treated with 10 *µ*M and 50 *µ*M cisplatin; ^#^*p* < 0.001 versus squirrel RTECs treated with 0 *µ*M cisplatin (control), and mouse RTECs treated with 10 *µ*M and 50 *µ*M cisplatin; ^$^*p* < 0.001 versus mouse RTECs treated with 50 *µ*M cisplatin, *n* = 3). Supplementary Figure S3: Akt1 shRNA-treated squirrel RTECs have significantly reduced expression of Akt1. Akt1 deficient squirrel RTECs treated with cisplatin treated have decreased protein expression of pAkt (ser473), pBAD (ser136), and increased expression of cleaved caspase-3 versus controls and wild type squirrel RTECs treated with cisplatin. (i) Akt1 (^*∗*^*p* < 0.0001versus Akt1 deficient squirrel RTECs treated with 0 *µ*M cisplatin (control) or 10 *µ*M and 50 *µ*M cisplatin; ^#^*p* < 0.01 versus squirrel RTECs treated with 50 *µ*M cisplatin, *n* = 3). (ii) pAkt (ser473) (^*∗*^*p* < 0.0001versus wild type and Akt1 deficient squirrel RTECs treated with 0 *µ*M cisplatin (controls) and Akt1 deficient squirrel RTECs treated with 10 *µ*M and 50 *µ*M cisplatin; ^#^*p* < 0.0001 versus Akt1 deficient squirrel RTECs treated with 0 *µ*M cisplatin (control), *n* = 3). (iii) pBAD (ser136) (^*∗*^*p* < 0.01versus wild type and Akt1 deficient squirrel RTECs treated with 0 *µ*M cisplatin (controls), wild type squirrel RTECs treated with 10 *µ*M cisplatin, and Akt1 deficient squirrel RTECs treated with 10 *µ*M and 50 *µ*M cisplatin; ^#^*p* < 0.001 versus wild type and Akt1 deficient squirrel RTECs treated with 0 *µ*M cisplatin (controls) and Akt1 deficient squirrel RTECs treated with 10 *µ*M and 50 *µ*M cisplatin; ^$^*p* < 0.0001 versus wild type and Akt1 deficient squirrel RTECs treated with 0 *µ*M cisplatin (controls) and Akt1 deficient squirrel RTECs treated with 10 *µ*M cisplatin; ^*∗∗*^*p* < 0.01 versus wild type and Akt1 deficient squirrel RTECs treated with 0 *µ*M cisplatin (controls), *n* = 3). (iv) CC3 (^*∗*^*p* < 0.0001versus wild type and Akt1 deficient squirrel RTECs treated with 0 *µ*M cisplatin (controls), wild type squirrel RTECs treated with 10 *µ*M and 50 *µ*M cisplatin, and Akt1 deficient squirrel RTECs treated with 10 *µ*M cisplatin; ^#^*p* < 0.001 versus wild type and Akt1 deficient squirrel RTECs treated with 0 *µ*M cisplatin (controls) *n* = 3). Supplementary Figure S4: reduced expression of XIAP in squirrel RTECs treated with XIAP siRNA. Cisplatin and XIAP siRNA-treated squirrel RTECs have decreased protein expression of XIAP and increased expression of cleaved caspase-3 compared to controls and wild type cisplatin-treated squirrel RTECs. (i) XIAP (^*∗*^*p* < 0.0001versus wild type and XIAP deficient squirrel RTECs treated with 0 *µ*M cisplatin (controls) and XAIP deficient squirrel RTECs treated with 10 *µ*M and 50 *µ*M cisplatin; ^#^*p* < 0.001 versus XIAP deficient squirrel RTECs treated with 0 *µ*M cisplatin (control), *n* = 3). (ii) CC3 (^*∗*^*p* < 0.0001versus wild type and XIAP deficient squirrel RTECs treated with 0 *µ*M cisplatin (controls), wild type squirrel RTECs treated with 10 *µ*M cisplatin, and XAIP deficient squirrel RTECs treated with 10 *µ*M and 50 *µ*M cisplatin; #*p* < 0.01 versus wild type and XIAP deficient squirrel RTECs treated with 0 *µ*M cisplatin (controls), *n* = 3).
