# Supplementary material for: Renal Tubular Cells from Hibernating Squirrels are Protected against Cisplatin Induced Apoptosis
Source: Int J Nephrol. 2020 Aug 4;2020:6313749. doi: 10.1155/2020/6313749 (PMC7424393; doi:10.1155/2020/6313749)
Supplement: Supplementary Materials — Supplementary Figure S1: cleaved caspase-3 protein expression is increased in mouse RTECs treated with 10 µM and 50 µM cisplatin compared to control mouse RTECs, control squirrel RTECs, and squirrel RTECs treated with 10 µM and 50 µM cisplatin (∗p < 0.0001 versus mouse and squirrel RTECs treated with 0 µM cisplatin (controls), and squirrel RTECs treated with 10 µM and 50 µM cisplatin, n = 3). Supplementary Figure S2: pAkt (ser473), pBAD (ser136), and XIAP protein expression are increased in squirrel RTECs on treatment with 10 µM and 50 µM cisplatin versus control squirrel RTECs. In contrast, mouse RTECs have reduced expression of pAkt (ser473), pBAD (ser136), and XIAP with cisplatin treatment compared to control mouse RTECs. (i) Akt1 (∗p < 0.01versus squirrel RTECs treated with 50 µM cisplatin, n = 3). (ii) pAkt (ser473) (∗p < 0.001versus mouse and squirrel RTECs treated with 0 µM cisplatin (controls), squirrel RTECs treated with 10 µM cisplatin and mouse RTECs treated with 10 µM and 50 µM cisplatin; #p < 0.0001 versus squirrel RTECs treated with 0 µM cisplatin (control) and mouse RTECs treated with 50 µM cisplatin, n = 3). (iii) pBAD (ser136) (∗p < 0.0001versus mouse and squirrel RTECs treated with 0 µM cisplatin (controls), squirrel RTECs treated with 10 µM cisplatin, and mouse RTECs treated with 10 µM and 50 µM cisplatin; #p < 0.0001 versus mouse and squirrel RTECs treated with 0 µM cisplatin (controls), and mouse RTECs treated with 10 µM and 50 µM cisplatin; $p < 0.0001 versus mouse RTECs treated with 0 µM cisplatin (control), and mouse RTECs treated with 10 µM and 50 µM cisplatin, n = 3). (iv) XIAP (∗p < 0.0001versus mouse and squirrel RTECs treated with 0 µM cisplatin (controls), squirrel, and mouse RTECs treated with 10 µM and 50 µM cisplatin; #p < 0.001 versus squirrel RTECs treated with 0 µM cisplatin (control), and mouse RTECs treated with 10 µM and 50 µM cisplatin; $p < 0.001 versus mouse RTECs treated with 50 µM cisplatin, n = 3). Supplementary Figure S3 [file 6313749.f1.docx]

**Supplementary Figure S1**

**
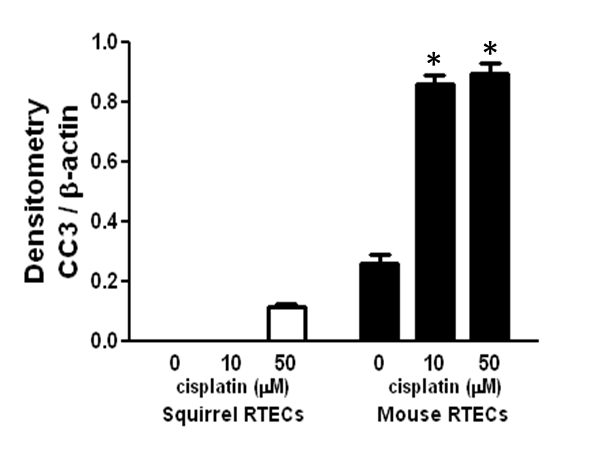
**

**Supplementary FigureS 1:** Cleaved caspase-3 protein expression is increased in mouse RTECs treated with 10 µM and 50 µM cisplatin compared to control mouse RTECs, control squirrel RTECs and squirrel RTECs treated with 10 µM and 50 µM cisplatin (*p <0.0001 vs. mouse and squirrel RTECs treated with 0 µM cisplatin (controls), and squirrel RTECs treated with 10 µM and 50 µM cisplatin, n=3).

**Supplementary Figure S2**

**
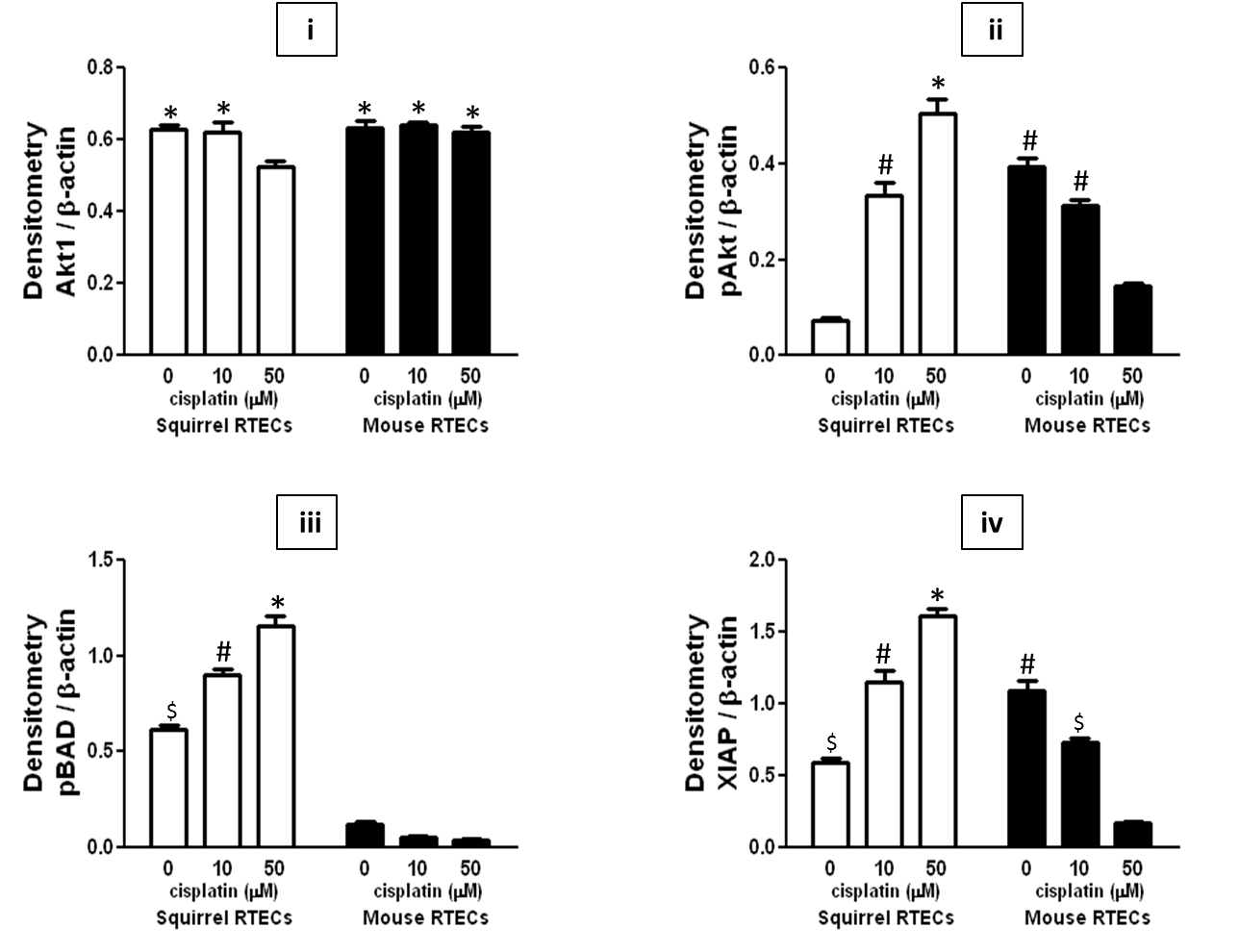
**

**Supplementary Figure S2:** pAkt (ser473), pBAD (ser136) and XIAP protein expression is increased in squirrel RTECs on treatment with 10 µM and 50 µM cisplatin vs. control squirrel RTECs. In contrast mouse RTECs have reduced expression of pAkt (ser473), pBAD (ser136) and XIAP with cisplatin treatment compared to control mouse RTECs. **i)** Akt1 (*p <0.01 vs. squirrel RTECs treated with 50 µM cisplatin, n=3). **ii)** pAkt (ser473) (*p <0.001 vs.. mouse and squirrel RTECs treated with 0 µM cisplatin (controls), squirrel RTECs treated with 10 µM cisplatin and mouse RTECs treated with 10 µM and 50 µM cisplatin; ^#^p <0.0001 vs. squirrel RTECs treated with 0 µM cisplatin (control) and mouse RTECs treated with 50 µM cisplatin, n=3). **iii)** pBAD (ser136) (*p <0.0001 vs. mouse and squirrel RTECs treated with 0 µM cisplatin (controls), squirrel RTECs treated with 10 µM cisplatin and mouse RTECs treated with 10 µM and 50 µM cisplatin; ^#^p <0.0001 vs. mouse and squirrel RTECs treated with 0 µM cisplatin (controls), and mouse RTECs treated with 10 µM and 50 µM cisplatin; ^$^p <0.0001 vs. mouse RTECs treated with 0 µM cisplatin (control), and mouse RTECs treated with 10 µM and 50 µM cisplatin, n=3). **iv)** XIAP (*p <0.0001 vs. mouse and squirrel RTECs treated with 0 µM cisplatin (controls), squirrel and mouse RTECs treated with 10 µM and 50 µM cisplatin; ^#^p <0.001 vs. squirrel RTECs treated with 0 µM cisplatin (control), and mouse RTECs treated with 10 µM and 50 µM cisplatin; ^$^p <0.001 vs. mouse RTECs treated with 50 µM cisplatin, n=3).

**Supplementary Figure S3**


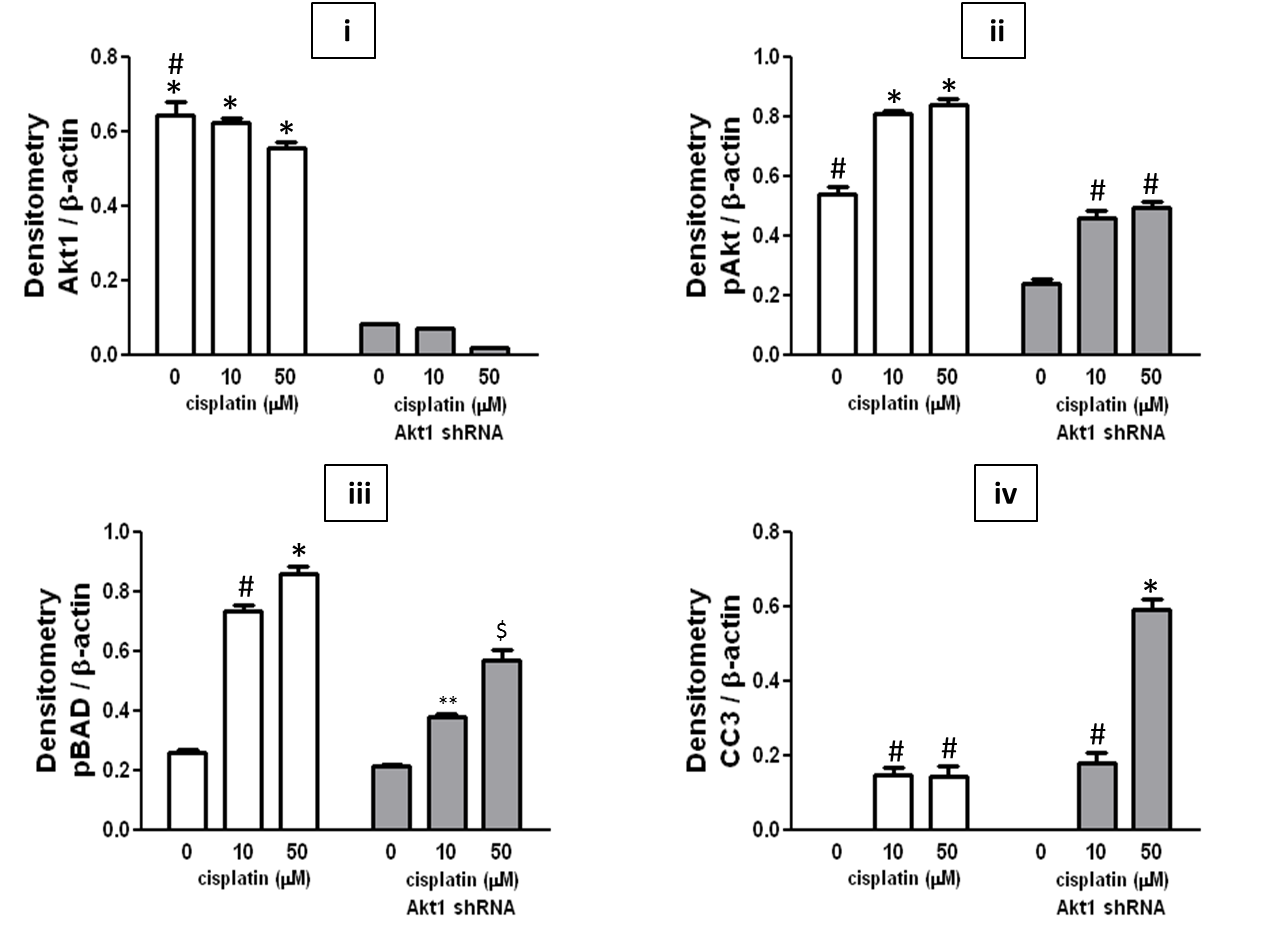


**Supplementary Figure S3:** Akt1 shRNA treated squirrel RTECs has significantly reduced expression of Akt1. Akt1 deficient squirrel RTECs treated with cisplatin treated have decreased protein expression of pAkt (ser473), pBAD (ser136) and increased expression of cleaved caspase-3 vs. controls and wild type squirrel RTECs treated with cisplatin. **i)** Akt1 (*p <0.0001 vs. Akt1 deficient squirrel RTECs treated with 0 µM cisplatin (control) or 10 µM and 50 µM cisplatin; ^#^p <0.01 vs. squirrel RTECs treated with 50 µM cisplatin, n=3). **ii)** pAkt (ser473) (*p <0.0001 vs. wild type and Akt1 deficient squirrel RTECs treated with 0 µM cisplatin (controls) and Akt1 deficient squirrel RTECs treated with 10 µM and 50 µM cisplatin; ^#^p <0.0001 vs. Akt1 deficient squirrel RTECs treated with 0 µM cisplatin (control), n=3). **iii)** pBAD (ser136) (*p <0.01 vs. wild type and Akt1 deficient squirrel RTECs treated with 0 µM cisplatin (controls), wild type squirrel RTECs treated with 10 µM cisplatin and Akt1 deficient squirrel RTECs treated with 10 µM and 50 µM cisplatin; ^#^p <0.001 vs. wild type and Akt1 deficient squirrel RTECs treated with 0 µM cisplatin (controls) and Akt1 deficient squirrel RTECs treated with 10 µM and 50 µM cisplatin; ^$^p <0.0001 vs. wild type and Akt1 deficient squirrel RTECs treated with 0 µM cisplatin (controls) and Akt1 deficient squirrel RTECs treated with 10 µM cisplatin; ^**^p <0.01 vs. wild type and Akt1 deficient squirrel RTECs treated with 0 µM cisplatin (controls), n=3). **iv)** CC3 (*p <0.0001 vs. wild type and Akt1 deficient squirrel RTECs treated with 0 µM cisplatin (controls), wild type squirrel RTECs treated with 10 µM and 50 µM cisplatin and Akt1 deficient squirrel RTECs treated with 10 µM cisplatin; ^#^p <0.001 vs. wild type and Akt1 deficient squirrel RTECs treated with 0 µM cisplatin (controls) n=3).

**Supplementary Figure S4**


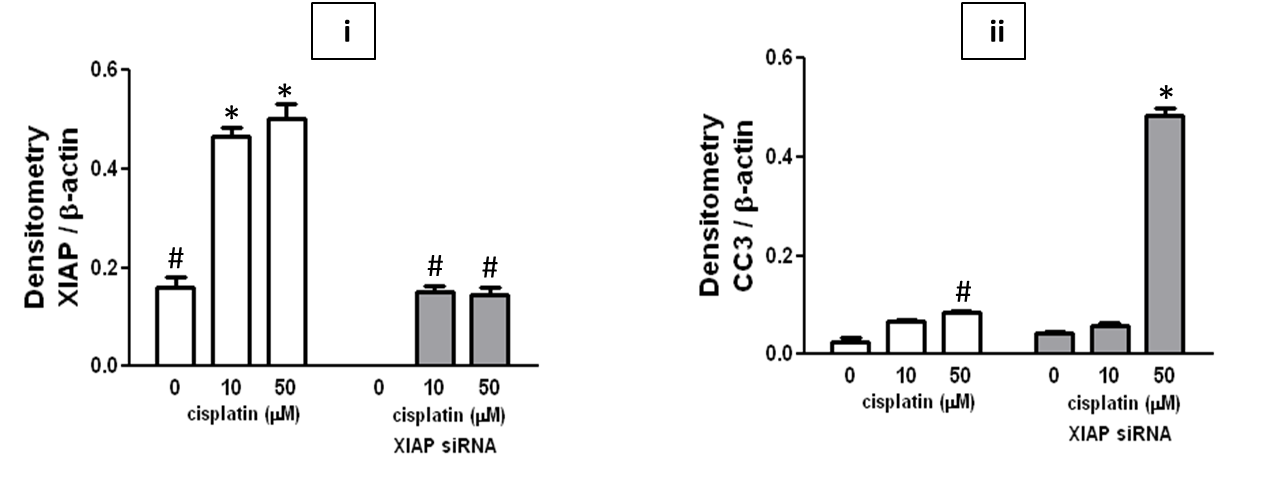


**Supplementary Figure S4:** Reduced expression of XIAP in squirrel RTECs treated with XIAP siRNA. Cisplatin and XIAP siRNA treated squirrel RTECs have decreased protein expression of XIAP and increased expression of cleaved caspase-3 compared to controls and wild type cisplatin treated squirrel RTECs. **i)** XIAP (*p <0.0001 vs. wild type and XIAP deficient squirrel RTECs treated with 0 µM cisplatin (controls) and XAIP deficient squirrel RTECs treated with 10 µM and 50 µM cisplatin; ^#^p <0.001 vs. XIAP deficient squirrel RTECs treated with 0 µM cisplatin (control), n=3). **ii)** CC3 (*p <0.0001 vs. wild type and XIAP deficient squirrel RTECs treated with 0 µM cisplatin (controls), wild type squirrel RTECs treated with 10 µM cisplatin and XAIP deficient squirrel RTECs treated with 10 µM and 50 µM cisplatin; ^#^p <0.01 vs. wild type and XIAP deficient squirrel RTECs treated with 0 µM cisplatin (controls), n=3).
